# Circulating Plasma Cells as a Biomarker to Predict Newly Diagnosed Multiple Myeloma Prognosis: Developing Nomogram Prognostic Models

**DOI:** 10.3389/fonc.2021.639528

**Published:** 2021-03-05

**Authors:** Qianwen Cheng, Li Cai, Yuyang Zhang, Lei Chen, Yu Hu, Chunyan Sun

**Affiliations:** ^1^Institute of Hematology, Wuhan Union Hospital, Tongji Medical College, Huazhong University of Science and Technology, Wuhan, China; ^2^Collaborative Innovation Center of Hematology, Huazhong University of Science and Technology, Wuhan, China

**Keywords:** newly diagnosed multiple myeloma, circulating plasma cells, biomarker, nomograms, prognosis

## Abstract

**Background:** To investigate the prognostic value of circulating plasma cells (CPC) and establish novel nomograms to predict individual progression-free survival (PFS) as well as overall survival (OS) of patients with newly diagnosed multiple myeloma (NDMM).

**Methods:** One hundred ninetyone NDMM patients in Wuhan Union Hospital from 2017.10 to 2020.8 were included in the study. The entire cohort was randomly divided into a training (*n* = 130) and a validation cohort (*n* = 61). Univariate and multivariate analyses were performed on the training cohort to establish nomograms for the prediction of survival outcomes, and the nomograms were validated by calibration curves.

**Results:** When the cut-off value was 0.038%, CPC could well distinguish patients with higher tumor burden and lower response rates (*P* < 0.05), and could be used as an independent predictor of PFS and OS. Nomograms predicting PFS and OS were developed according to CPC, lactate dehydrogenase (LDH) and creatinine. The C-index and the area under receiver operating characteristic curves (AUC) of the nomograms showed excellent individually predictive effects in training cohort, validation cohort or entire cohort. Patients with total points of the nomograms ≤ 60.7 for PFS and 75.8 for OS could be defined as low-risk group and the remaining as high-risk group. The 2-year PFS and OS rates of patients in low-risk group was significantly higher than those in high-risk group (*p* < 0.001).

**Conclusions:** CPC is an independent prognostic factor for NDMM patients. The proposed nomograms could provide individualized PFS and OS prediction and risk stratification.

## Introduction

Multiple myeloma (MM) is a malignant plasma cell disorder with wide variation in clinical progression and prognosis, which would be estimated 32,270 new cases and 12,830 deaths in American in 2020 (1). Results from the Surveillance, Epidemiology, and End Results database showed an increase in 10–20-year relative survival of MM patients between 2002–2006 and 2012–2016 (2). In China, the incidence of MM kept increasing from 2006 to 2016, but the mortality remained stable between 2014 and 2016 after the increase from 2006 to 2014, which might due to the development of new therapeutic approaches such as bortezomib, lenalidomide, and hematopoietic stem cell transplantation (3–5). The prognosis of MM is highly heterogeneous, with some patients surviving for more than 10 years and others for only a few months (6). The prognostic factors and staging systems of MM are changing with the development of detection techniques and treatment strategies. The first widely used staging system of MM was the Durie-Salmon (D-S) staging system in 1975 (7). Later, with the development of new treatment methods, the International Staging System (ISS) was established in 2005 (8). With increasing attention paid to cytogenetic features, Mayo Stratification of Myeloma and Risk-Adapted Therapy (mSMART) 2007, updated mSMART 2013, mSMART 3.0 in 2018 and the International Myeloma Working Group (IMWG) 2016 staging system all recommended using cytogenetic abnormalities (CA) as the risk stratification criteria for MM patients (9–12). The Revised International Staging System (R-ISS) proposed in 2015 have incorporated CA and lactate dehydrogenase (LDH) (13).

Circulating plasma cells (CPC) has been found to have prognostic significance in MM patients for decades (14), and the technology to detect CPC has changed from slide-base immunofluorescence assay to multi-parameter flow cytometry (MFC). Subsequent studies have shown that CPC has prognostic significance both in patients with newly diagnosed MM (NDMM) (15), refractory/relapsed MM (16), smoldering MM (17), and MM patients who have undergone autologous hematopoietic stem cell transplantation (ASCT) (18, 19). But there is no conclusion on the optimal cut-off value of CPC at present, and few studies have reported how we should apply the results of CPC in combination with other predictors to predict survival in MM patients in clinical application.

Nomogram is constructed on the basis of multivariate regression models (such as Cox and logistic regression models), which can transform complex regression equations into simple and visual graphics, and making the results of prediction model more readable and valuable for use (20). When predicting the probability of individual events, nomogram is more quickly, intuitively, and accurately, compared with the traditional clinical staging systems (21, 22). Therefore, nomogram has been increasingly used in clinical practice, especially in predicting the recurrence, metastasis or death of cancer patients (22–26). In this retrospective study, we used seven-color MFC to evaluate CPC in peripheral blood of NDMM patients. Then we explored the correlation of CPC with the clinical features and prognosis of NDMM patients, and established nomograms to predict individual progression-free survival (PFS) and overall survival (OS) based on CPC.

## Patients and Methods

### Patients

**Figure 1:**
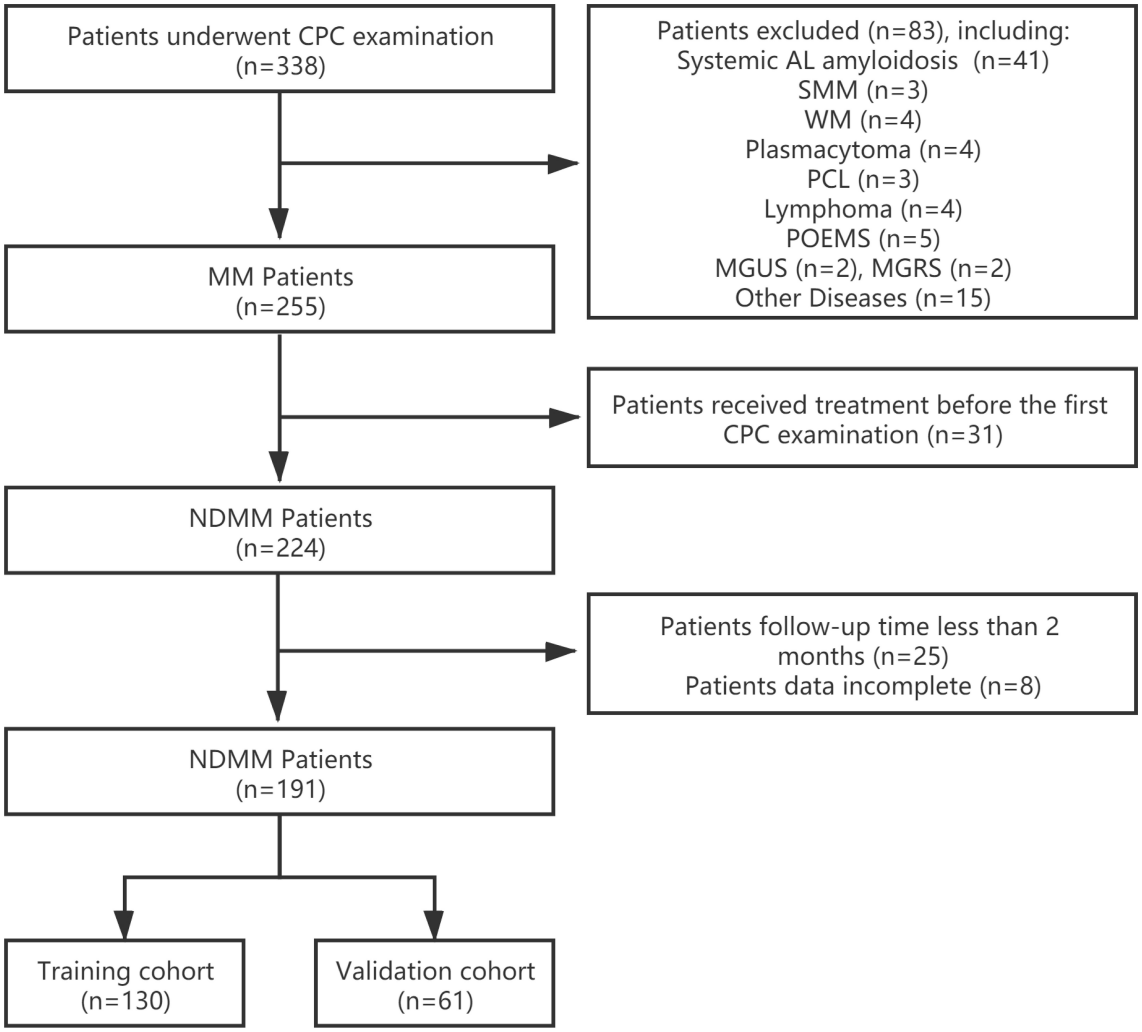
Patient flow diagram. CPC, circulating plasma cells; MM, multiple myeloma; NDMM, newly diagnosed multiple myeloma; systemic AL amyloidosis, systemic light chain amyloidosis; SMM, smoldering multiple myeloma; WM, Waldenström macroglobulinemia; PCL, plasma cell leukemia; POEMS, POEMS Syndrome; MGUS, monoclonal gammopathy of undetermined significance; MGRS, monoclonal gammopathy of renal significance.

We conducted a retrospective study on 191 consecutive patients with NDMM treated in Wuhan Union hospital of China between 2017.10 and 2020.8. The International Myeloma Working Group (IMWG) criteria was used to assess the diagnosis and treatment response. Patients with diseases such as Waldenström macroglobulinemia, lymphoma, plasma cell leukemia, systemic light chain amyloidosis (AL amyloidosis), and MM patients who previously had received chemotherapy were excluded. The patient's flow diagram was in Figure 1. The clinical information was collected retrospectively by reviewing the patients' medical records. PFS was calculated from the beginning of first-line chemotherapy until the date of disease progression, death, or the last date the patient was known to be free of disease progression. OS was calculated from the beginning of first-line chemotherapy until death or the last date the patient was known to be alive. The degree of myeloma bone disease (MBD) was assessed using radiographic methods. This study has been approved by the Ethics Committee of Tongji Medical College of Huazhong University of Science and Technology and followed the principles of the Declaration of Helsinki.

### Quantification of CPC

CPC levels at the time of diagnosis were detected and quantified by two-tube, seven-color MFC. Peripheral blood mononuclear cells isolated by Ficoll gradient were analyzed by flow cytometric analyses and stained with antibodies to CD38, CD138, CD45, CD56, CD19, and cytoplasmic kappa and lambda immunoglobulin light chains. Samples were examined on a BD FACS Canto flow cytometer (BD Biosciences) and data were analyzed by BD FACS Diva 8.0 (BD Biosciences). The sensitivity of clonal CPC was more than 0.001% (10^6^ cells were collected per tube), and the results of CPC were reported as a percentage of total mononuclear cells.

### Construction of the Nomograms

The 191 NDMM patients were randomly divided into a training cohort of 130 patients and a validation cohort of 61 patients with a ratio of 7:3 through a random number list generated by SPSS. The training cohort was used to establish the PFS and OS nomograms. In the training cohort, the following clinical features were assessed to identify predictors of survival: age, sex, D-S stage, ISS stage, R-ISS stage, extramedullary myeloma, first line therapy regimens, ASCT, CA, clonal bone marrow plasma cells (BMPC), LDH, albumin (ALB), β2-microglobulin (β2-MG), hypersensitive C-reactive protein (hs-CRP), CPC, calcium, creatinine, hemoglobin, and monoclonal protein (M-protein). According to the R-ISS in 2015, IMWG in 2016 and mSMART 3.0 in 2018, high-risk CA include t (4; 14), gain (1q), del (17p), t (14; 16) as well as t (14; 20) (11–13). Univariate analysis of potential risk factors for PFS and OS was performed using the Cox proportional hazards regression model. Variables in the univariate analysis with *p* < 0.10 were chosen for multivariate Cox proportional hazard regression to identify the independent prognostic factors. Based on the results of the multivariate Cox regression analyses, nomogram models to predict PFS and OS for NDMM patients were formulated.

### Validation of the Nomograms

The prognostic performance of the nomogram was validated by measuring discrimination and calibration in training, validation and entire cohort. The predictive power of the nomogram was assessed by C-index (concordance index), and a higher C-index indicates a better ability to discriminate patients among different survival outcomes. The calibration plots generated by 1,000 bootstraps resampling reflect the agreement between observed outcomes and predicted probabilities. The total point of nomogram for PFS and OS was calculated for each patient. In addition, receiver operating characteristic (ROC) curve analysis were conducted to further evaluate the predictive performance of the nomogram total point for PFS and OS, and the discriminative power of different models were evaluated by calculating the area under the ROC curve (AUC).

### Statistical Analysis

Baseline continuous variables were presented as mean and standard deviation for normally distributed data or as median and inter-quartile range (IQR) for non-normal data, and categorical variables were presented as counts and percentages. Continuous variables were compared using the independent Student's *t*-test or Mann-Whitney *U*-test, and categorical data were compared using χ^2^-test or Fisher's exact test. ROC curve analysis was performed to determine the optimal cut-off values of continuous variables based on maximum Youden index. PFS and OS rates were estimated by the Kaplan-Meier method and compared between groups by the log-rank test. A 2-sided *p* < 0.05 was considered statistically significant. Statistical analyses were conducted using SPSS version 23.0 (IBM Corp). Kaplan-Meier survival curves and scatter plots were generated by GraphPad Prism (Version 8.0.2, GraphPad Software Inc.). ROC curves were plotted using MedCalc (Version 18.2.1). The nomograms and calibration curves were formulated with the R software (Version 3.6.1, R Project for Statistical Computing, Vienna, Austria).

## Results

### Clinical Characteristics

The baseline characteristics of the training and validation cohorts are presented in Table 1. A total of 191 patients were included in the analysis, whose average age was 59.0 ± 9.8 years (range: 30–88 years), with 58 (30.4%) patients of 65 year-old or older. Among these patients, there were 105 (55%) men and 86 (45%) women, with a median CPC of 0.006% (0.000–0.120%), and a median clonal BMPC of 19% (7–40%). In total, 160 (83.8%) patients received proteasome inhibitors (PIs)-containing regimens (bortezomib and carfilzomib), 31 (16.2%) received immunomodulatory drugs (IMiDs)-based regimens (lenalidomide and thalidomide) as first line therapy regimen. The patients were randomly divided into a training cohort of 130 patients and a validation cohort of 61 patients. There were no significant differences between the two groups of patients in age, sex, clonal bone marrow plasma cells, CPC, hemoglobin, albumin, creatinine, β2-MG, LDH, hs-CRP, extramedullary myeloma, MM type, high-risk CA, D-S, ISS, R-ISS, first line therapy regimens, ASCT, or response rates.

**Table 1:** Patients' baseline characteristics.

**Characteristics**	**Training cohort (*n* = 130)**	**Validation cohort (*n* = 61)**	***P***
Age, years, mean (SD)	59 (9)	59 (11)	0.736
Sex, *n* (%)			0.884
Male	71 (54.6)	34 (55.7)	
Female	59 (45.4)	27 (44.3)	
Clonal bone marrow plasma cells, %, Median (IQR)	17 (7–41)	21 (8–39)	0.333
CPC, %, median (IQR)	0.005 (0.000–0.122)	0.009 (0.000–0.135)	0.718
CPC status, *n* (%)			0.748
≤ 0.038%	82 (63.1)	37 (60.7)	
>0.038%	48 (34.9)	24 (39.3)	
Hemoglobin, g/L, mean (SD)	98 (25)	93 (26)	0.184
Albumin, g/L, mean (SD)	37.0 (6.6)	36 (5)	0.104
Creatinine, μmol/L, median (IQR)	80.0 (61.8–119.2)	91.9(61.0–223.7)	0.234
β2-MG, mg/L, median (IQR)	4.1 (2.7–7.7)	5.4 (2.9–10.8)	0.222
LDH, U/L, median (IQR)	194 (160–250)	184 (146–271)	0.884
hs-CRP, mg/L, median (IQR)	0.87 (0.00–9.04)	2.72 (0.21–8.31)	0.222
Extramedullary myeloma, *n* (%)	12 (9.2)	3 (4.9)	0.457
Type, *n* (%)			0.070
IgG	60 (46.2)	34 (55.7)	
IgA	35 (26.9)	7 (11.5)	
IgD	9 (6.9)	8 (13.1)	
Other	26 (20.0)	12 (19.7)	
High-risk CA, *n* (%)	58 (44.6)	34 (55.7)	0.151
D-S, *n* (%)			0.361
I	15 (11.5)	5 (8.2)	
II	28 (21.5)	9 (14.8)	
III	87 (66.9)	47 (77.0)	
ISS, *n* (%)			0.103
I	37 (28.5)	13 (21.3)	
II	48 (36.9)	17 (27.9)	
III	45 (34.6)	31 (50.8)	
R-ISS, *n* (%)			0.466
I	29 (22.3)	11 (18.0)	
II	71 (54.6)	31 (50.8)	
III	30 (23.1)	19 (31.1)	
First line therapy regimens, *n* (%)			0.705
PIs-containing regimens	108 (83.1)	52 (85.2)	
IMiDs-based regimens	22 (16.9)	9 (14.8)	
ASCT, *n* (%)	8 (6.2)	1 (1.6)	0.314
Response rates, *n* (%)			
VGPR or better	67 (51.5)	29 (47.5)	0.606
PR or better	98 (75.4)	48 (78.7)	0.616

*CPC, circulating plasma cells; β2-MG, β2-microglobulin; LDH, lactate dehydrogenase; hs-CRP, hypersensitive C-reactive protein; High-risk CA, High-risk Cytogenetic abnormalities, D-S, Durie-Salmon staging system; ISS, International Staging System; R-ISS, Revised-International Staging System; PIs, proteasome inhibitors; IMiDs, immunomodulatory drugs; ASCT, autologous hematopoietic stem cell transplantation; VGPR, very good partial response; PR, partial response*.

### Survival of Training and Validation Cohorts

With a median follow-up duration of 11 months (range: 2–36 months), the 2-year PFS rate was 45.8% and the 2-year OS rate was 77.2% among all patients. The median follow-up duration was 11 months (range: 2–36 months) in training cohort and 12 months (range: 2–36 months) in validation cohort, respectively (*P* = 0.178). Kaplan-Meier analysis of the NDMM patients showed that the 2-year PFS rate was 42.1 and 50.8% for the training cohort and the validation cohort, respectively (*P* = 0.539), while the 2-year OS rate was 78.0 and 75.7%, respectively (*P* = 0.886).

### The Relationship Among CPC, Patient Survival and Clinical Characteristics

**Figure 2:**
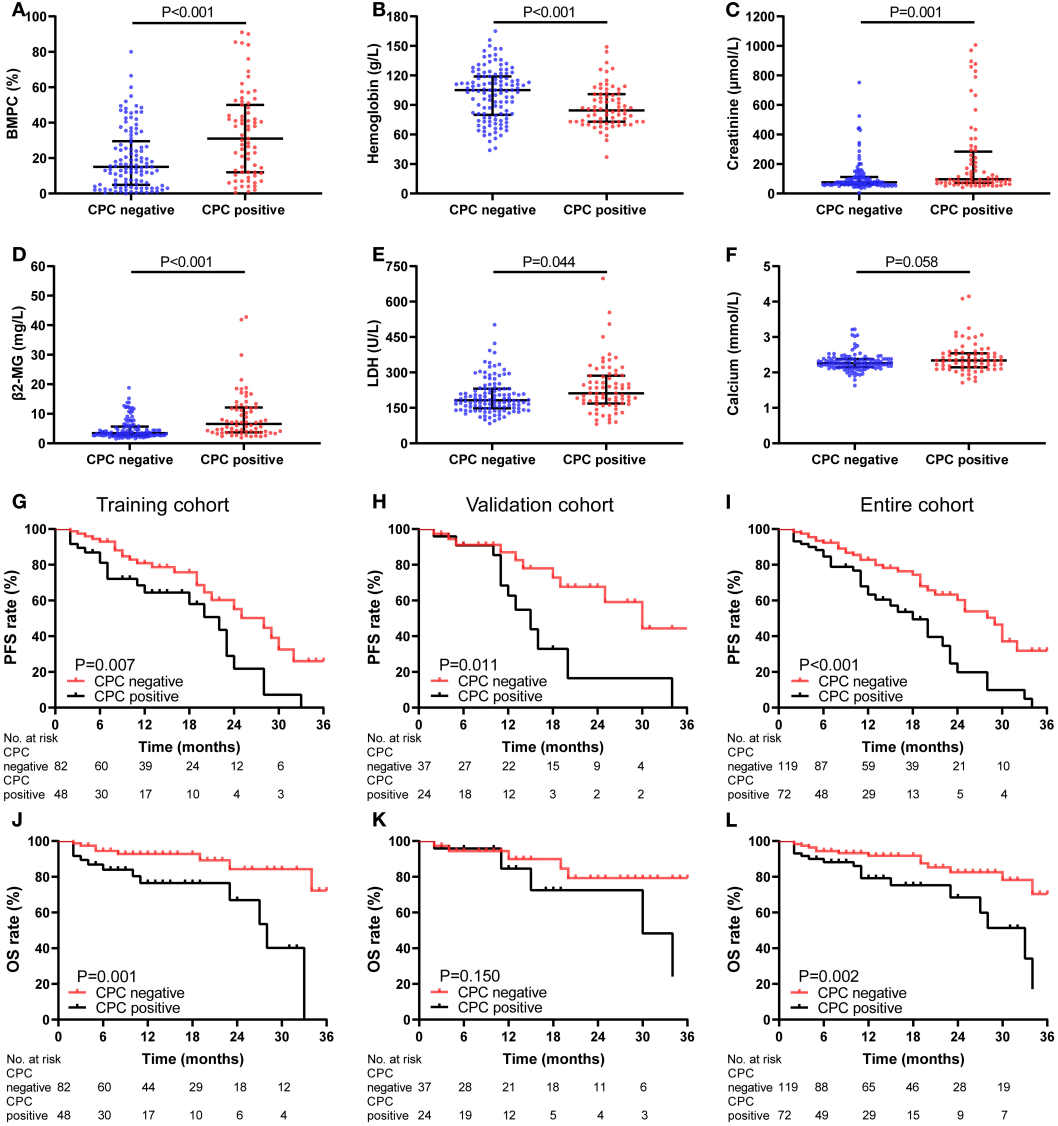
Characteristics and Kaplan-Meier curves of CPC negative (≤0.038%) (*n* = 119) and positive (>0.038%) (*n* = 72) group. **(A–F)** Comparison of BMPC, hemoglobin, creatinine, β2-MG, LDH, and calcium between CPC negative and positive group (*P* < 0.05). The PFS **(G–I)** and OS **(J–L)** curves for patients in training cohort **(G,J)**, validation cohort **(H,K)**, and entire cohort **(I,L)**.

CPC were detected in 113 patients (59.2%) with a median of 0.070% (range, 0.001–9.670%). We tested different cut-off values for CPC, and used 0.038% as the cut-off value for subsequent analysis because of the largest Youden index for OS (training cohort and entire cohort: 0.298 and 0.223). We compared patients with undetectable CPC (*n* = 78) and those with detectable CPC less than 0.038% (*n* = 41), and found that the baseline features, response rates and survival showed no differences between the two group of patients (*p* > 0.05) (Supplementary Table 1). So, the grade of CPC was performed as follows: CPC negative group included 119 patients who had <0.038% or undetectable CPC, CPC positive group included patients who had more than 0.038% CPC. Statistically significant differences were found in markers of high disease burden including BMPC, hemoglobin, creatinine, β2-MG, LDH, and MBD between patients of CPC negative and positive group (*P* < 0.05). CPC positive was significantly associated with D-S (*p* = 0.046), ISS (*p* < 0.001), and R-ISS stage (*p* < 0.001). The response rate of CPC positive group (37.5% ≥ very good partial response and 68.1% ≥ partial response) was lower than of negative group (58.0% ≥ very good partial response and 81.5% ≥ partial response) (*P* < 0.05) (Table 2; Figures 2A–F). In the training cohort and entire cohort, the PFS and OS rates of CPC negative group were better than those of CPC positive group (2-year PFS rate of training cohort: 55.2 vs. 21.7%, *p* = 0.007; 2-year OS rate of training cohort: 84.3 vs. 66.9%, *p* = 0.001; 2-year PFS rate of entire cohort: 60.3 vs. 19.8%, *p* < 0.001; 2-year OS rate of entire cohort: 82.5 vs. 68.4%, *p* = 0.002). In the validation cohort, PFS rate of CPC negative group was better than that of CPC positive group (2-year PFS rate: 67.6 vs. 16.4%, *p* = 0.011), but there was no significant difference in OS rate between the two groups (2-year OS rate: 79.3 vs. 72.5%, *p* = 0.150) (Figures 2G–L).

**Table 2:** The relationship between CPC and clinical characteristics.

**Characteristics**	**CPC negative (≤0.038%) (*n* = 119)**	**CPC positive (>0.038%) (*n* = 72)**	***P***
Age, years, mean (SD)	58 (9)	60 (11)	0.211
Sex, *n* (%)			0.439
Male	68 (57.1)	37 (51.4)	
Female	51 (42.9)	35 (48.6)	
Clonal bone marrow plasma cells, %, median (IQR)	15 (5–31)	31 (12–50)	<0.001
Hemoglobin, g/L, mean (SD)	101 (26)	88 (21)	<0.001
Albumin, g/L, mean (SD)	36.8 (6.3)	36.2 (6.1)	0.433
Creatinine, μmol/L, median (IQR)	75.8 (60.2–111.6)	99.2 (72.1–292.4)	0.001
β2-MG, mg/L, median (IQR)	3.5 (2.6–6.0)	7.0 (3.7–12.6)	<0.001
LDH, U/L, median (IQR)	182 (148–236)	207 (166–275)	0.044
hs-CRP, mg/L, median (IQR)	1.01 (0.00–6.98)	1.95 (0.00–11.50)	0.279
Calcium, mmol/L, median (IQR)	2.26 (2.15–2.38)	2.34 (2.15–2.54)	0.058
Extramedullary myeloma, *n* (%)	11 (9.2)	4 (5.6)	0.358
Myeloma bone disease, *n* (%)			0.032
0–3	55 (46.2)	22 (30.6)	
>3	64 (53.8)	50 (69.4)	
Type, *n* (%)			0.861
IgG	59 (49.6)	35 (48.6)	
IgA	28 (23.5)	14 (19.4)	
IgD	10 (8.4)	7 (9.7)	
Other	22 (18.5)	16 (22.2)	
High-risk CA, *n* (%)	51 (42.9)	41 (56.9)	0.059
D-S, *n* (%)			0.046
I	17 (14.3)	3 (4.2)	
II	25 (21.0)	12 (16.7)	
III	77 (64.7)	57 (79.2)	
ISS, *n* (%)			<0.001
I	42 (35.3)	8 (11.1)	
II	45 (37.8)	20 (27.8)	
III	32 (26.9)	44 (61.1)	
R-ISS, *n* (%)			<0.001
I	33 (27.7)	7 (9.7)	
II	68 (57.1)	34 (47.2)	
III	18 (15.1)	31 (43.1)	
First line therapy regimens, *n* (%)			0.349
PIs-containing regimens	102 (85.7)	58 (80.6)	
IMiDs-based regimens	17 (14.3)	14 (19.4)	
ASCT, *n* (%)	7 (5.9)	2 (2.8)	0.326
Response rates, *n* (%)			
VGPR or better	69 (58.0)	27 (37.5)	0.006
PR or better	97 (81.5)	49 (68.1)	0.034

*CPC, circulating plasma cells; β2-MG, β2-microglobulin; LDH, lactate dehydrogenase; hs-CRP, hypersensitive C-reactive protein; High-risk CA, High-risk Cytogenetic abnormalities, D-S, Durie-Salmon staging system; ISS, International Staging System; R-ISS, Revised-International Staging System; PIs, proteasome inhibitors; IMiDs, immunomodulatory drugs; ASCT, autologous hematopoietic stem cell transplantation; VGPR, very good partial response; PR, partial response*.

### Nomogram of PFS for NDMM

#### Development and Validation of a Nomogram of PFS for NDMM

**Figure 3:**
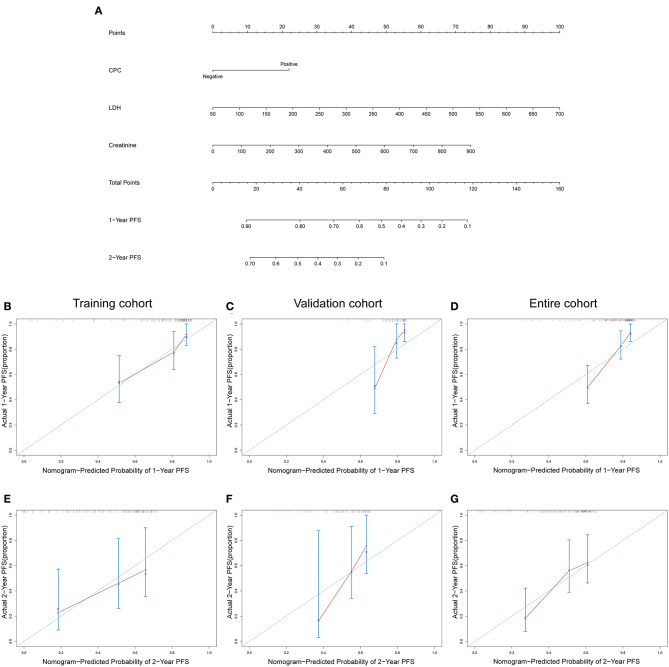
**(A)** Nomogram of PFS for patients with NDMM. Calibration curves for predicting 1-year and 2-year PFS in training cohort **(B,E)**, validation cohort **(C,F)**, and entire cohort **(D,G)**. CPC negative: ≤0.038%; CPC positive: >0.038%.

As shown in Table 3, univariate Cox regression analysis showed that CPC, creatinine, LDH, β2-MG and high-risk CA were correlated with PFS, and multivariate analysis identified CPC, creatinine and LDH as independent predictors of PFS in NDMM patients. The above three independent predictors of PFS were integrated into a PFS rate estimation nomogram (Figure 3A). The method to estimate PFS rates based on nomogram: the value of the creatinine, LDH and CPC is located on each variable axis, and then draw an upward line to the point axis to determine the number of points corresponding to each variable value. The sum of these points is located on the total point axis, and then a downward line is drawn from the total points axis to the survival axis to get the 1-year and 2-year PFS rates. The C-index of the nomogram in discriminating PFS in the training cohort was 0.738 (95% CI: 0.643–0.832), and the calibration plots showed good agreement between the predicted PFS and the observed PFS rate (Figures 3B,E). In the validation cohort and the entire cohort, the C-index of the nomogram were 0.687 (95% CI: 0.549–0.824) and 0.716 (95% CI: 0.637–0.795). The calibration plots also showed good agreement between predictions and actual observations in both validation cohort (Figures 3C,F) and entire cohort (Figures 3D,G).

**Table 3:** Univariate and multivariate Cox analysis for OS and PFS in patients with NDMM in training cohort.

**Variable**	**Univariate**		**Multivariate**	
	**HR (95% CI)**	***P***	**HR (95% CI)**	***P***
**PFS**				
β2-MG	1.034 (1.004–1.066)	0.028	0.949 (0.893–1.009)	0.094
CPC	2.157 (1.202–3.871)	0.010	2.047 (1.101–3.807)	0.024
Creatinine	1.003 (1.002–1.004)	<0.001	1.004 (1.002–1.006)	<0.001
LDH	1.004 (1.002–1.007)	0.001	1.005 (1.002–1.008)	0.001
High-risk CA	1.720 (0.963–3.075)	0.067	1.627 (0.885–2.990)	0.117
**OS**				
CPC	4.125 (1.626–10.470)	0.003	3.394 (1.253–9.192)	0.016
Creatinine	1.004 (1.002–1.005)	<0.001	1.004 (1.002–1.006)	<0.001
LDH	1.004 (1.001–1.008)	0.008	1.005 (1.001–1.009)	0.022
β2-MG	1.041 (1.008–1.076)	0.015	0.965 (0.903–1.031)	0.294
Hemoglobin	0.981 (0.962–1.001)	0.057	0.993 (0.971–1.016)	0.541
High-risk CA	1.968 (0.824–4.696)	0.127		

*PFS, progression-free survival; OS, overall survival; CPC, circulating plasma cells; β2-MG, β2-microglobulin; LDH, lactate dehydrogenase; High-risk CA, High-risk Cytogenetic abnormalities*.

#### The C-Index and AUC of Nomogram and D-S, ISS, and R-ISS Staging System for PFS

**Figure 4:**
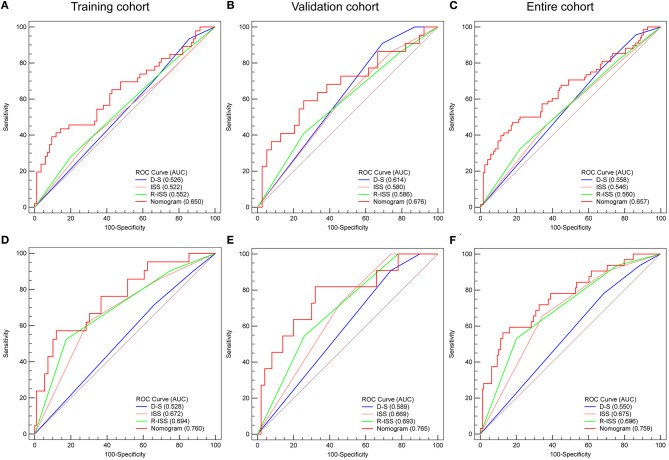
Area under the ROC curves of nomogram, D-S, ISS, and R-ISS stage of PFS **(A–C)** and OS **(D–F)** in training cohort **(A,D)**, validation cohort **(B,E)**, and entire cohort **(C,F)**.

The results showed that the C-index of the nomogram in the training cohort, validation cohort, and entire cohort was 0.738, 0.687, and 0.716, respectively. In the training cohort, validation cohort, and entire cohort, the C-index of D-S was 0.532, 0.604, and 0.553, the C-index of ISS was 0.608, 0.596, and 0.601, and the C-index of R-ISS was 0.621, 0.601, and 0.612. The AUC of the nomogram and the D-S, ISS and R-ISS staging systems was shown in Figures 4A–C.

### Nomogram of OS for NDMM

#### Development and Validation of a Nomogram of OS for NDMM

**Figure 5:**
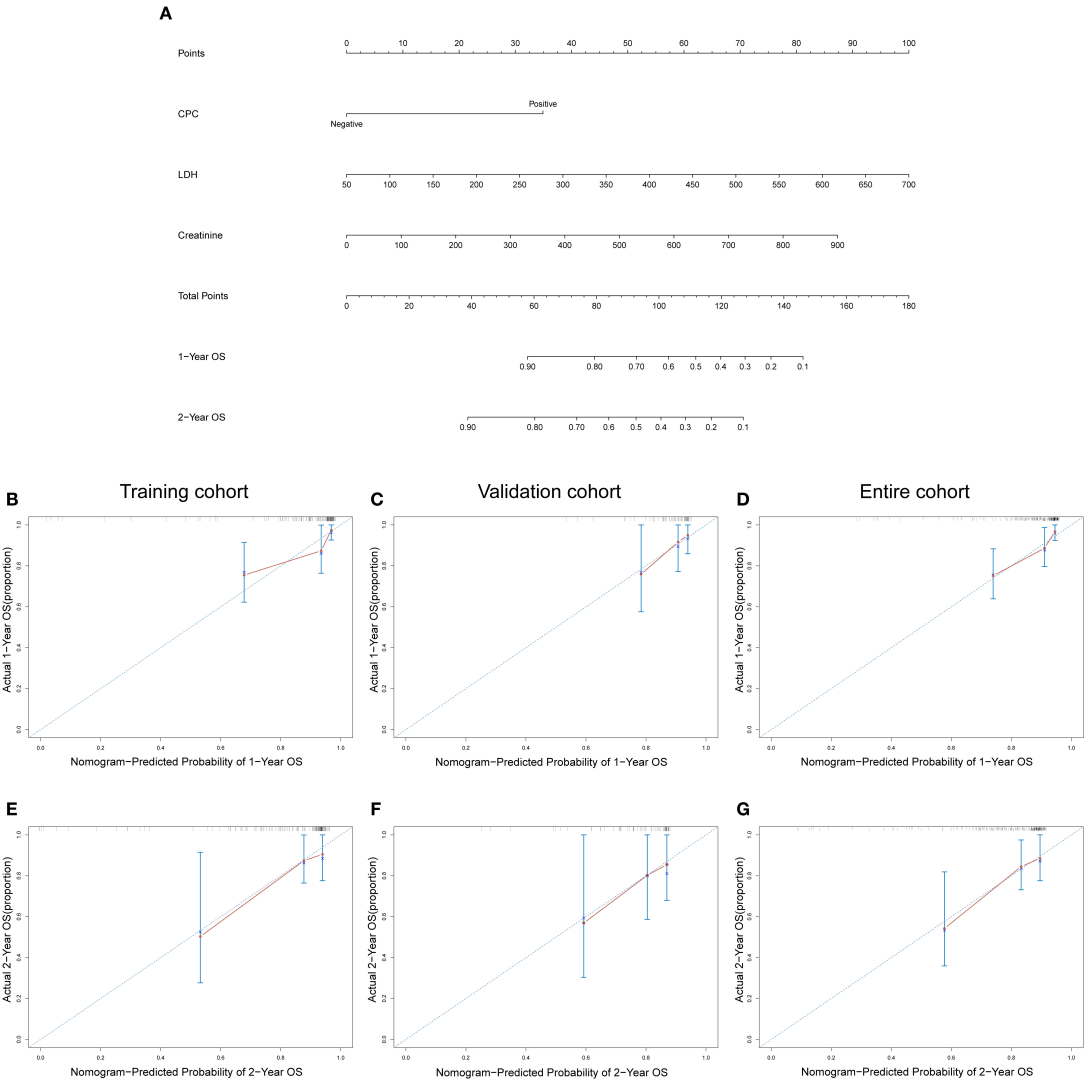
**(A)** Nomogram of OS for patients with NDMM. Calibration curves for predicting 1-year and 2-year OS in training cohort **(B,E)**, validation cohort **(C,F)**, and entire cohort **(D,G)**. CPC negative: ≤0.038%; CPC positive: >0.038%.

The results of univariate and multivariate Cox regression analysis based on pretreatment data are shown in Table 3. Univariate analysis showed that CPC, creatinine, LDH, β2-MG, and hemoglobin were correlated with OS. Multivariate analysis identified CPC, creatinine and LDH as independent predictors for OS of NDMM patients. Then, the above three independent predictors of OS were integrated into an OS rate estimation nomogram (Figure 5A). The nomogram of OS is used in a similar way to the nomogram of PFS. The C-index of the nomogram in discriminating OS in the training cohort was 0.802 (95% CI: 0.679–0.926), and the calibration plots showed good agreement between the predicted OS and the observed OS rate (Figures 5B,E). In the validation cohort and the entire cohort, the C-index of the nomogram were 0.722 (95% CI: 0.512–0.931) and 0.766 (95% CI: 0.655–0.878). The calibration plots also showed good agreement between predictions and actual observations both in the validation cohort (Figures 5C,F) and the entire cohort (Figures 5D,G).

#### The C-Index and AUC of Nomogram and D-S, ISS, and R-ISS Staging System for OS

The results showed that the C-index of the nomogram in the training cohort, validation cohort, and entire cohort was 0.802, 0.722, and 0.766, respectively. In the training cohort, validation cohort, and entire cohort, the C-index of D-S was 0.514, 0.603, and 0.538, the C-index of ISS was 0.688, 0.650, and 0.681, and the C-index of R-ISS was 0.705, 0.669, and 0.697. The AUC of the nomogram and the D-S, ISS and R-ISS staging system was shown in Figures 4D–F.

### Distinguishing High- and Low-Risk Patients Based on the Nomogram Total Points

**Figure 6:**
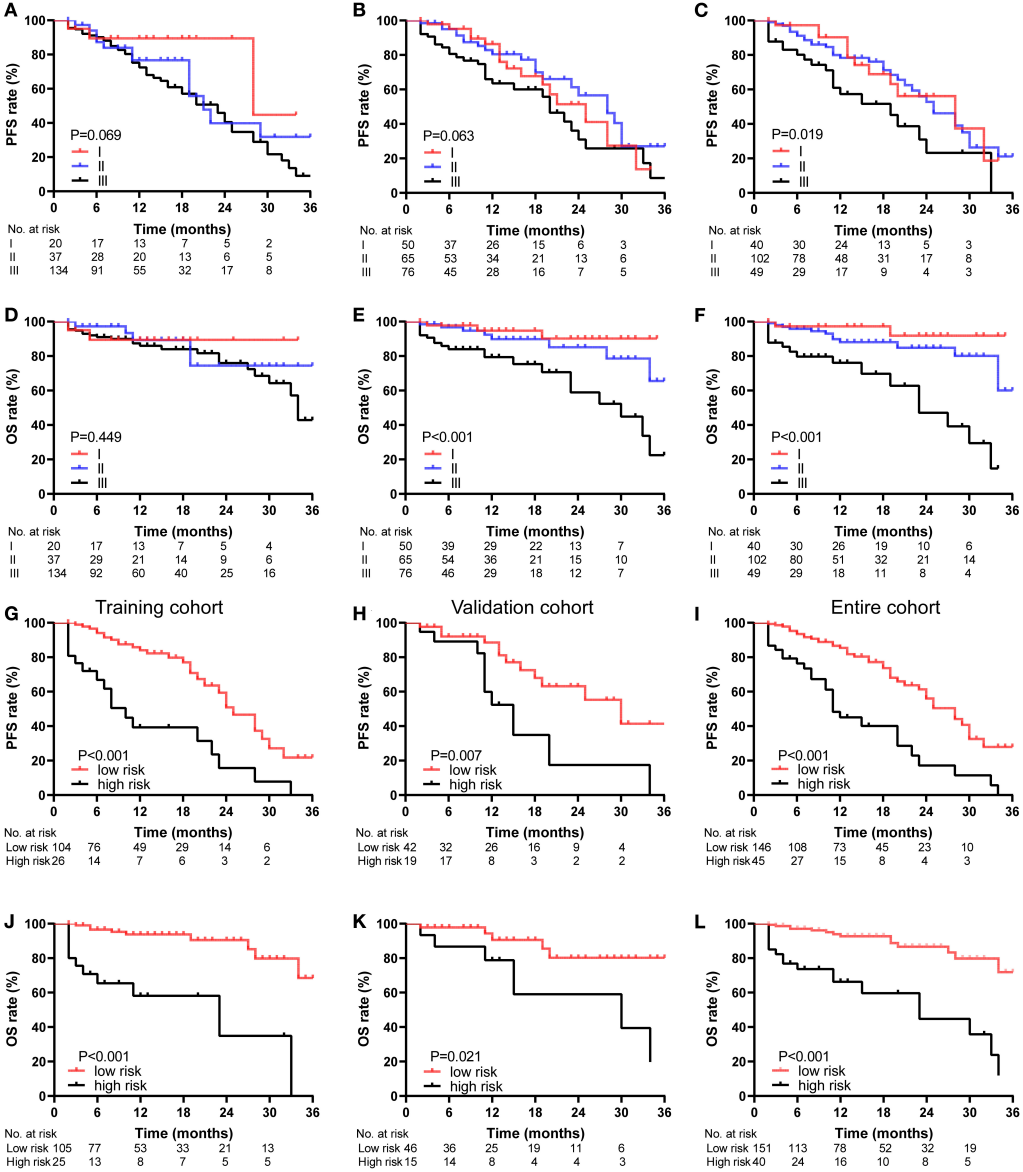
Kaplan-Meier survival curves of the NDMM patients, categorized by different staging systems. PFS **(A–C)** and OS **(D–F)** curve of the D-S **(A,D)**, ISS **(B,E)** and R-ISS **(C,F)** stage in entire cohort. PFS **(G–I)** and OS **(J–L)** curve of the nomogram stage in training cohort **(G,J)**, validation cohort **(H,K)**, and entire cohort **(I,L)**.

As shown in Figure 6, for PFS of patients in entire cohort, all of the three existing staging systems (D-S, ISS, and R-ISS staging system) cannot distinguish well the prognosis of patients among stage I, II, and III (Figures 6A–C). And for OS of them, the D-S cannot well distinguish the prognosis of patients among stage I, II, and III (Figure 6D), the ISS cannot well distinguish the prognosis of patients between stage I and II (Figure 6E), and the R-ISS showed good prognostic stratification for the patients among stages I, II, and III (Figure 6F). Each patient was given a total point for PFS and a total point for OS based on the 2 nomogram models. With the threshold of 60.7 for PFS and 75.8 for OS, the nomogram score respectively stratified patients from the 3 cohorts into high-risk and low-risk groups. For PFS, 45 patients were assigned to the high-risk group (>60.7) and 146 to the low-risk group (≤60.7). Eighty percent (36/45) of patients in the high-risk group and 84.9% (124/146) of patients in the low-risk group received PIs-containing regimens as first line therapy (*p* = 0.433). The 2-year PFS rate of patients in low-risk group was significantly higher than that in high-risk group (all patients: 56.0 vs 17.2%, *p* < 0.001; HR: 3.241, 95% CI: 1.992–5.272) (Figures 6G–I). For OS, there were 40 patients in the high-risk group (>75.8) and 151 patients in the low-risk group (≤75.8). 82.5% (33/40) of the high-risk group and 84.1% (127/151) of the low-risk group received PI-containing regimens as first line therapy (*p* = 0.807). The difference of 2-year OS rate between low-risk group and high-risk group was statistically significant (all patients: 86.6 vs. 44.7%, *p* < 0.001; HR: 5.651, 95% CI: 2.807–11.379) (Figures 6J–L). According to the survival analysis of PFS and OS, the nomogram showed excellent prediction ability when divided patients into high-risk and low-risk groups (*p* < 0.05 in training cohort, validation cohort, and entire cohort) (Figures 6G–L).

## Discussion

The prognostic significance of CPC measured by MFC has been well established in MM patients at the time of diagnosis (15, 27–29), and the objective of this analysis was to develop and validate a CPC based individual prognostic nomogram for patients with NDMM. The C-index of the nomogram for PFS were 0.738 (95% CI: 0.643–0.832) in training cohort, 0.687 (95% CI: 0.549–0.824) in validation cohort and 0.716 (95% CI: 0.637–0.795) in entire cohort. The C-index of the nomogram for OS were 0.802 (95% CI: 0.679–0.926) in training cohort, 0.722 (95% CI: 0.512–0.931) in validation cohort and 0.766 (95% CI: 0.655–0.878) in entire cohort. The C-indexes and ROC curves demonstrated that nomograms showed excellent individually predictive effects in predicting PFS and OS of patients with NDMM in training cohort, validation cohort or entire cohort. Patients with total points of the nomograms ≤60.7 for PFS and 75.8 for OS could be defined as low-risk group with a 2-year PFS rate of 56.0% and a 2-year OS rate of 86.6%, while patients with total points of the nomograms >60.7 for PFS and 75.8 for OS could be defined as high-risk group with a 2-year PFS rate of 17.2% and a 2-year OS rate of 44.7. Our results suggest that when predicting the individual PFS and OS rates, nomogram is a quickly, intuitively and accurately tool, and the risk stratification based on nomogram shows excellent performance.

Our nomograms for PFS and OS both included CPC, creatinine, and LDH. All these factors had been found to be associated with advanced myeloma in previous studies. Serum creatinine was a commonly used indicator to evaluate renal function in daily work and had been included in D-S staging system and IMWG updated criteria for the diagnosis of MM (7, 30). Thanks to the advent of new drugs, the survival of patients with renal insufficiency has improved considerably over the past decade. However, patients with severe renal insufficiency, especially the elderly, still have a high risk of early death (31, 32). High serum LDH level have been shown to be a marker of aggressive myeloma and short survival, and have been included in the R-ISS (13, 32, 33). Our results support that serum creatinine and LDH as independent predictors of PFS and OS in patients with NDMM (Table 3).

The cut-off value of CPC is different among studies, ranged from 10 CPC per 50,000 events (0.02%) to 400 CPC per 150,000 events (0.267%) (15, 27, 34–37). Differences in cut-off values may be attributed to difference of patient populations, detection techniques, and treatment regimens. Since most of the relevant reports are single-center studies, it is not clear whether these cut-off values are applicable to the prognosis of all MM patients, so there is no consensus on the optimal cut-off value of CPC. But in different studies, patients in high CPC group showed highly proliferative disease in terms of higher BMPC, LDH, M-protein, β2-MG and high risk CA, higher D-S, ISS and R-ISS stage, lower hemoglobin and albumin, and shorter survival time (27, 36, 38–40). Our study found that when the cut-off value was 0.038%, CPC could be used as an independent predictor of PFS and OS, and could well distinguish patients with higher tumor burden, more aggressive tumor status and lower response rates as well (Table 2; Figure 2). It is important for its clinical application to determine the optimal cut-off value of CPC, and our research may provide a better choice for the optimal cut-off value of CPC.

Although there have been several nomogram prediction models for NDMM patients in previous studies, to our knowledge, this is the first nomogram based on CPC, and the first nomogram to predict PFS in NDMM patients (41–43). The nomograms we developed in this study to predict the PFS and OS rates of NDMM patients showed excellent individually predictive effects. Moreover, the nomograms showed greater predictive ability in PFS and OS than the commonly used MM staging models such as the D-S, ISS and R-ISS staging system. Compared with other studies, our nomogram is simpler, more intuitive and easier to apply in clinical practice.

Although our nomogram performed well in predicting PFS and OS in NDMM patients, there were some limitations in this study. First, as with other retrospective studies, data bias exists. Second, our nomograms were developed and validated using single-center data without external validation using data from other research centers. Third, a limited number of subjects were included in this study, and the median follow-up time was only 11 months. Fourth, due to low economic level, low health insurance coverage of the transplant drugs, low educational level, and low acceptance of standardized and holistic treatment of the disease, only 7% of the eligible patients received ASCT in our research. So, the results in our study need to be further corroborated by data from prospective and other institutions, and the verification of the nomograms requires a larger sample size and a longer follow-up time to get more convincing results when implemented in clinical practice.

## Conclusions

In conclusion, we developed a nomogram to predict 1-year, 2-year PFS and a nomogram to predict 1-year, 2-year OS as prognostic tools for patients with NDMM. Our results suggest that the nomograms can individually and accurately predict patient prognosis and risk stratification.

## Data Availability Statement

The raw data supporting the conclusions of this article will be made available by the authors, without undue reservation.

## Ethics Statement

The studies involving human participants were reviewed and approved by the Ethics Committee of Tongji Medical College of Huazhong University. The patients/participants provided their written informed consent to participate in this study.

## Author Contributions

QC collected, analyzed the data, and wrote the paper. LCa, YZ, and LCh researched the literature and revised the paper. CS and YH conceived and designed the study, analyzed the data, and wrote the paper. All authors reviewed the paper and approved the final manuscript.

## Conflict of Interest

The authors declare that the research was conducted in the absence of any commercial or financial relationships that could be construed as a potential conflict of interest.
