# Supplementary material for: Circulating Plasma Cells as a Biomarker to Predict Newly Diagnosed Multiple Myeloma Prognosis: Developing Nomogram Prognostic Models
Source: Front Oncol. 2021 Mar 5;11:639528. doi: 10.3389/fonc.2021.639528 (PMC7973368; doi:10.3389/fonc.2021.639528)
Supplement: Supplementary file 1 [file Table_1.DOCX]

Supplementary Table S1. The comparison between undetectable CPC and detectable CPC less than 0.038%.

| **Characteristics** | **CPC undetectable**  **(n=78)** | **CPC low**  **(0~0.038%) (n=41)** | **P** | |
| --- | --- | --- | --- | --- |
| Age, years, mean (SD) | 57 (9) | 60 (10) | 0.151 | |
| Sex, n (%) |  |  | 0.230 | |
| Male | 47 (60.3) | 20 (48.8) |  | |
| Female | 31 (39.7) | 21 (51.2) |  | |
| Clonal bone marrow plasma cells, %, Median (IQR) | 16 (5-31) | 15 (6-23) | 0.585 | |
| Hemoglobin, g/L, mean (SD) | 100 (25) | 97 (25) | 0.338 | |
| Albumin, g/L, mean (SD) | 36.7 (6.4) | 36.8 (5.8) | 0.832 | |
| Creatinine, μmol/L, Median (IQR) | 75.6 (59.9-110.8) | 76.5 (55.7-119.3) | 0.788 | |
| β2-MG, mg/L, Median (IQR) | 3.5 (2.7-5.8) | 3.5 (2.7-7.4) | 0.485 | |
| LDH, U/L, Median (IQR) | 182 (148-237) | 170 (142-231) | 0.324 | |
| hs-CRP, mg/L, Median (IQR) | 0.91 (0.00-7.02) | 0.71 (0.00-7.57) | 0.419 | |
| Calcium, mmol/L, Median (IQR) | 2.26 (2.15-2.38) | 2.26 (2.15-2.38) | 0.496 | |
| Extramedullary myeloma, n (%) | 5 (6.4) | 6 (14.6) | 0.141 | |
| Myeloma bone disease, n (%) |  |  | 0.238 | |
| 0-3 | 33 (42.3) | 22 (53.7) |  | |
| >3 | 45 (57.7) | 19 (46.3) |  | |
| Type, n (%) |  |  | 0.474 | |
| IgG | 35 (44.9) | 24 (58.5) |  | |
| IgA | 19 (24.4) | 9 (22.0) |  | |
| IgD | 8 (10.3) | 2 (4.9) |  | |
| other | 16 (20.5) | 6 (14.6) |  | |
| High-risk CA, n (%) | 35 (44.9) | 16 (39.0) | 0.540 | |
| D-S, n (%) |  |  | 0.298 | |
| I | 9 (11.5) | 8 (19.5) |  | |
| II | 19 (24.4) | 6 (14.6) |  | |
| III | 50 (64.1) | 27 (65.9) |  | |
| ISS, n (%) |  |  | 0.608 | |
| I | 30 (38.5) | 12 (29.3) |  | |
| II | 28 (35.9) | 17 (41.5) |  | |
| III | 20 (25.6) | 12 (29.3) |  | |
| R-ISS, n (%) |  |  | 0.810 | |
| I | 23 (29.5) | 10 (24.4) |  | |
| II | 43 (55.1) | 25 (61.0) |  | |
| III | 12 (15.4) | 6 (14.6) |  | |
| First line therapy regimens, n (%) |  |  | 0.937 | |
| PIs-containing regimens | 67 (85.9) | 35 (85.4) |  | |
| IMiDs-based regimens | 11 (14.1) | 6 (14.6) |  | |
| ASCT, n (%) | 7 (9) | 0 (0) | 0.094 | |
| Response rates, n (%) |  |  | |  |
| VGPR or better | 45 (57.7) | 24 (58.5) | | 0.929 |
| PR or better | 64 (82.1) | 33 (80.5) | 0.835 | |
| 2-year survival, % |  |  |  | |
| PFS | 60.0% | 63.5% | 0.733 | |
| OS | 82.2% | 83.7% | 0.727 | |

CPC, circulating plasma cells; β2-MG, β2-microglobulin; LDH, lactate dehydrogenase; hs-CRP, hypersensitive C-reactive protein; High-risk CA, High-risk Cytogenetic abnormalities, D-S, Durie-Salmon staging system; ISS, International Staging System; R-ISS, Revised-International Staging System; PIs, proteasome inhibitors; IMiDs, immunomodulatory drugs; ASCT, autologous hematopoietic stem cell transplantation; VGPR, very good partial response; PR, partial response; PFS, progression-free survival, OS, overall survival.
